# Changes in drug sensitivity and anti-malarial drug resistance mutations over time among *Plasmodium falciparum* parasites in Senegal

**DOI:** 10.1186/1475-2875-12-441

**Published:** 2013-12-06

**Authors:** Daria Van Tyne, Baba Dieye, Clarissa Valim, Rachel F Daniels, Papa Diogoye Sène, Amanda K Lukens, Mouhamadou Ndiaye, Amy K Bei, Yaye Die Ndiaye, Elizabeth J Hamilton, Omar Ndir, Souleymane Mboup, Sarah K Volkman, Dyann F Wirth, Daouda Ndiaye

**Affiliations:** 1 Department of Immunology and Infectious Diseases, Harvard School of Public Health, Boston 02115, USA; 2 Laboratory of Parasitology and Mycology, Université Cheikh Anta Diop, BP5005, Dakar, Senegal; 3 Infectious Disease Initiative, Broad Institute, Cambridge 02141, USA; 4 Department of Human and Evolutionary Biology, Harvard University, Cambridge 02138, USA; 5 Laboratory of Bacteriology and Virology, Université Cheikh Anta Diop, Dakar BP5005, Senegal; 6 School of Nursing and Health Sciences, Simmons College, Boston 02115, USA

**Keywords:** Drug resistance monitoring, DAPI *ex vivo* assay, Malaria

## Abstract

**Background:**

Malaria treatment efforts are hindered by the rapid emergence and spread of drug resistant parasites. Simple assays to monitor parasite drug response in direct patient samples (*ex vivo*) can detect drug resistance before it becomes clinically apparent, and can inform changes in treatment policy to prevent the spread of resistance.

**Methods:**

Parasite drug responses to amodiaquine, artemisinin, chloroquine and mefloquine were tested in approximately 400 *Plasmodium falciparum* malaria infections in Thiès, Senegal between 2008 and 2011 using a DAPI-based *ex vivo* drug resistance assay. Drug resistance-associated mutations were also genotyped in *pfcrt* and *pfmdr1*.

**Results:**

Parasite drug responses changed between 2008 and 2011, as parasites became less sensitive to amodiaquine, artemisinin and chloroquine over time. The prevalence of known resistance-associated mutations also changed over time. Decreased amodiaquine sensitivity was associated with sustained, highly prevalent mutations in *pfcrt*, and one mutation in *pfmdr1* – Y184F – was associated with decreased parasite sensitivity to artemisinin.

**Conclusions:**

Directly measuring *ex vivo* parasite drug response and resistance mutation genotyping over time are useful tools for monitoring parasite drug responses in field samples. Furthermore, these data suggest that the use of amodiaquine and artemisinin derivatives in combination therapies is selecting for increased drug tolerance within this population.

## Background

*Plasmodium falciparum* malaria has an enormous public health impact, infecting millions and killing hundreds of thousands of people each year [1]. Drug resistance further magnifies the burden of this disease, as resistant malaria parasites have been selected by nearly every anti-malarial drug used to date. Reports of parasites with reduced susceptibility to artemisinin combination therapy (ACT) [2,3] underscore the importance of closely monitoring parasite drug responses and optimizing control strategies to quickly identify and prevent the spread of resistant parasites, particularly on the African continent [4].

Malaria drug resistance monitoring involves directly measuring parasite drug responses, or indirectly measuring the prevalence of resistance-associated mutations within a parasite population. *Ex vivo* drug resistance assays measure drug response in parasites taken directly from infected patients, without prior culture adaptation. These assays allow the components of combination therapies to be tested individually against parasites, and they can detect decreases in drug efficacy before resistance becomes clinically evident and widespread [5]. Many assays have been developed to test parasite drug resistance in both laboratory and field settings [6-13]. In addition, mutations in a number of parasite genetic loci have been shown to contribute to anti-malarial drug resistance, including *pfcrt* and *pfmdr1*, among others [14]. Monitoring the prevalence of these mutations consistently over several years can reveal trends in allele selection within a population over time, and can extend the therapeutic life of current and future treatments [15,16].

The motivation for this study was to ask whether the malaria parasites circulating in Thiès, Senegal were becoming more or less resistant to anti-malarial drugs over time, and whether changes in parasite drug response could be explained by known drug resistance-associated mutations. Drug use in Senegal changed from chloroquine monotherapy to sulphadoxine, pyrimethamine and amodiaquine in 2003, and again to ACT (predominantly artesunate-amodiaquine in Thiès) in 2006 [17]. Previous drug resistance monitoring efforts in Senegal have focused on directly testing parasite drug sensitivity [11,18], measuring the prevalence of resistance-associated mutations [19,20], or both. The aim of this study was to measure both parasite drug sensitivity and resistance mutation prevalence over time, in order to understand how parasites in Senegal may be changing in response to drug treatment.

## Methods

### Study population

Individuals seeking treatment for uncomplicated *P. falciparum* malaria at the Section de Lutte Antiparasitaire (SLAP) clinic in Thiès, Senegal, during the fall transmission seasons of 2008-2011 were tested for malaria infection by microscopy and rapid diagnostic test (RDT). *Plasmodium falciparum*-positive patients were eligible for screening if they met the criteria described below and the patient or legal guardian provided informed written consent or assent. Eligibility criteria were: patients older than two years, axillary temperature above 37.5°C or history of fever within the preceding 24 hours, infection with only *P. falciparum*, no recent anti-malarial drug use, and a haemoglobin level greater than 6 g/dL. Patients with symptoms of severe malaria were excluded and referred to the Thiès regional hospital for appropriate care. Study protocols and informed consent documents were approved by the Institutional Review Boards of the Senegal Ministry of Health IRB Committee and the Harvard School of Public Health (Senegal Protocol #16330; Harvard Protocol #P10256-127).

Among the 831 patients with uncomplicated malaria screened at the SLAP clinic between 2008 and 2011, a subset of 397 patient samples were tested for parasite drug response using the DAPI *ex vivo* assay (Table 1). The subset of patients that were tested was comparable to the larger set of screened patients with respect to demographic parameters (age, gender) and clinical characteristics (temperature, haematocrit, weight) with the exception of parasitaemia.

**Table 1:** **Clinical parameters in screened patients and the subset tested using the DAPI *ex vivo *** **assay**

	**All screened patients**	**DAPI tested patients**	***P***
Number	831	397	-
Gender (% male)	66	64	0.59
Age (years)	20 (15, 28)	20 (14, 26)	0.24
Weight (kg)	55 (42, 65)	55 (39, 65)	0.46
Temperature (°C)	38.2 (37.2, 39.7)	38.4 (37.3, 40.0)	0.25
Haematocrit (%)	38 (32, 40)	38 (32, 40)	0.79
Parasitaemia (%)	0.50 (0.20, 1.00)	0.61 (0.40, 1.10)	<.0001

Median values (with interquartile ranges) are reported for age, weight, temperature, haematocrit and parasitaemia. *P*-values were calculated using Pearson X^2^ for categorical variables, and Wilcox rank-sum test for continuous variables.

### Sample collection and DAPI ex vivo testing

From each subject, 5-10 mL venous blood were collected and processed on the same day. Approximately 1 mL of blood was spotted onto Whatman FTA™ filter paper cards for subsequent DNA extraction; the remaining blood was spun at 1,500 rpm for 10 minutes, plasma and buffy coat were removed, and infected red blood cells were washed twice with unsupplemented RPMI media. Aliquots of each sample were cryopreserved in Glycerolyte 57 (Fenwal) supplemented with AB^+^ serum, for subsequent culture adaptation and *in vitro* repeat drug testing.

Parasites were drug tested using the previously described DAPI *ex vivo* assay [11]. Briefly, 180 μL of supplemented RPMI media containing parasitized erythrocytes at 2% haematocrit were distributed into 96-well plates preloaded with 20 μL serial dilutions of amodiaquine (USP 1031004), artemisinin (Sigma A5430), chloroquine (Sigma C6628) and mefloquine (Sigma M2319). Drug concentrations ranged from less than 1 nM to greater than 1 μM and each plate included 6-8 negative control wells with media only. Plates contained two wells of each drug concentration, were prepared in a single batch, and were frozen prior to use. When possible, samples with parasitaemia greater than 1% were diluted into leukocyte-free donor O^+^ erythrocytes to a final plating parasitaemia of 0.4-1%.

Parasites were cultured 48-72 hours at 37°C under standard gas conditions (1% O_2_, 5% CO_2_, 94% N_2_) before addition of 4′,6-diamidino-2-phenylindole (DAPI) solution, as described previously [8,11]. Data were collected by measuring relative fluorescence units (RFUs) using a Fluoroskan plate reader (Thermo Scientific; ex 358 nm, em 461 nm). 3D7 parasites were tested on each batch of drug plates to control for batch variation, and there were no consistent trends toward increased resistance among parasites tested later each season, suggesting minimal batch degradation.

### DNA extraction, clonality of infection and HRM genotyping

DNA was extracted from 4-5 circular 6 mm punches of blood preserved on Whatman FTA™ filter paper cards using either a QIAmp DNA Blood Mini Kit (Qiagen) or a Maxwell DNA IQ Casework Sample Kit (Promega). Parasite genomic DNA was quantified by quantitative Real-Time PCR (qPCR) [21], and clonality of infection, defined as monoclonal or polyclonal, was assessed using the 24-SNP molecular barcode [21].

High resolution melt (HRM) technology was used to genotype a set of single nucleotide polymorphisms (SNPs) associated with reduced drug sensitivity [19]. Mutations were detected based on changes in DNA sequence; in the text, mutations are referred to by the corresponding amino acid changes. Briefly, 0.01 ng of parasite template, as quantified by qPCR [21], was used for each 5 μL reaction, which included 2.5× LightScanner Master Mix with LCGreen Plus dsDNA dye (BioFire Diagnostics, Inc.). HRM analysis and genotype determination was performed on a LightScanner-384 (BioFire Diagnostics, Inc.). The HRM method can determine genotypes from as little as 10 pg of parasite DNA and can detect mutant alleles present at less than 1% [19].

### Culture adaptation and in vitro drug testing

To assess whether *ex vivo* drug responses were reproducible *in vitro*, 16 parasite isolates derived from monoclonal infections collected in 2009 were culture adapted and re-tested *in vitro*. Culturing was conducted under standard conditions [22] with gentle shaking at 55 rpm. Parasites were *in vitro* drug tested against a panel of known anti-malarials using a standard hypoxanthine incorporation assay [7], or a SYBR Green I-based drug assay [23] with modifications for 384-well format.

### Calculation of IC_50_ values and data exclusion

Fluorescence data from drug assays were analysed using GraphPad Prism (San Diego, CA) through a four-parameter, log-logistic nonlinear regression of fluorescence intensity versus log_10_-transformed drug concentrations. To include control wells with no drug in the analysis, 1 nM was added to each concentration value. Dose-response curves were visually inspected for fit of the sigmoidal dose-response model. Among 397 patient samples tested using the DAPI *ex vivo* assay, 25 samples were considered assay failures due to no parasite growth or assay contamination and were excluded from further analysis. An additional two patient samples with a plating parasitaemia below 0.1% and 30 samples with a plating parasitaemia above 1.5% were excluded, because there was no clear association between plating parasitaemia and fluorescence intensity in the no-drug wells, perhaps due to insufficient growth or saturation. This left 340 patient samples from which parasite response to at least one anti-malarial drug was determined.

Drug curves that did not exhibit the standard sigmoidal dose-response shape were classified as either fitting an exponential or linear decay model, and had their IC_50_ values estimated through these alternative models, or were excluded. When IC_50_ values from technical replicates could not be estimated due to a single outlier point, this point was excluded.

### Data and statistical analysis

Dynamic range of the DAPI *ex vivo* assay was assessed by calculating the signal-to-noise ratio (SNR) and Z’-factor of each assay. SNR was measured by dividing fluorescence signal (RFUs) from no-drug wells by fluorescence signal from maximum drug wells. The median signal-to-noise ratio (SNR) among all assays was 3:1 (Interquartile Range = 2:1, 5:1). Z’-factor was calculated using the following equation: Z’ = 1- [(3 standard deviations of positive controls + 3 standard deviations of negative controls)/absolute difference between negative and positive controls] [24]. The median Z’-factor among all assays was 0.61; Z’-factors greater than 0 are considered acceptable, and Z’-factors greater than 0.5 are considered excellent [24]. Reliability of the DAPI *ex vivo* assay was measured by evaluating agreement between technical replicates in the untransformed scale using the intraclass correlation coefficient (ICC) for agreement. Only sigmoidal curves were analysed, to avoid biases due to lack of fitness of different IC_50_ curve-fitting models. For all drugs, mean differences in IC_50_ values between replicates were approximately zero, as expected. Except for a few outliers (fewer than five points for each drug), differences between replicates were small compared with the IC_50_ range of each drug.

Statistical analyses were performed in GraphPad Prism (v5.0d, San Diego, CA) and R-2.11.1. IC_50_ values measured *ex vivo* were compared to *in vitro* IC_50_ values from culture-adapted parasites by calculating the intraclass correlation coefficient (ICC) for consistency (R package *irr*), and by linear regression. To monitor population drug sensitivity, IC_50_ variations over time were measured through linear regression with log_10_-transformed IC_50_ values. Primary analysis focused on non-linear trends (using indicator variables for years) but results were confirmed by assessing linear trends. Multiple regression models were used to measure whether IC_50_ values changed significantly over time after accounting for the effect of potential confounders (clonality, haematocrit, parasitaemia, age, and temperature). Since parasites with reduced drug sensitivity may first arise in subpopulations that exhibit larger IC_50_ values, the 90^th^ percentile among all IC_50_ values is reported for each year. Changes in the prevalence of drug resistance markers over time were measured by Fisher-Hamilton exact test, and 95% confidence intervals for marker prevalence are based on the logit (R package *binom*). Finally, associations between IC_50_ values and the occurrence of drug resistance-associated mutations in *pfcrt* and *pfmdr1* were assessed through the Wilcoxon rank-sum test.

## Results

### DAPI ex vivo assay validation

**Figure 1:**
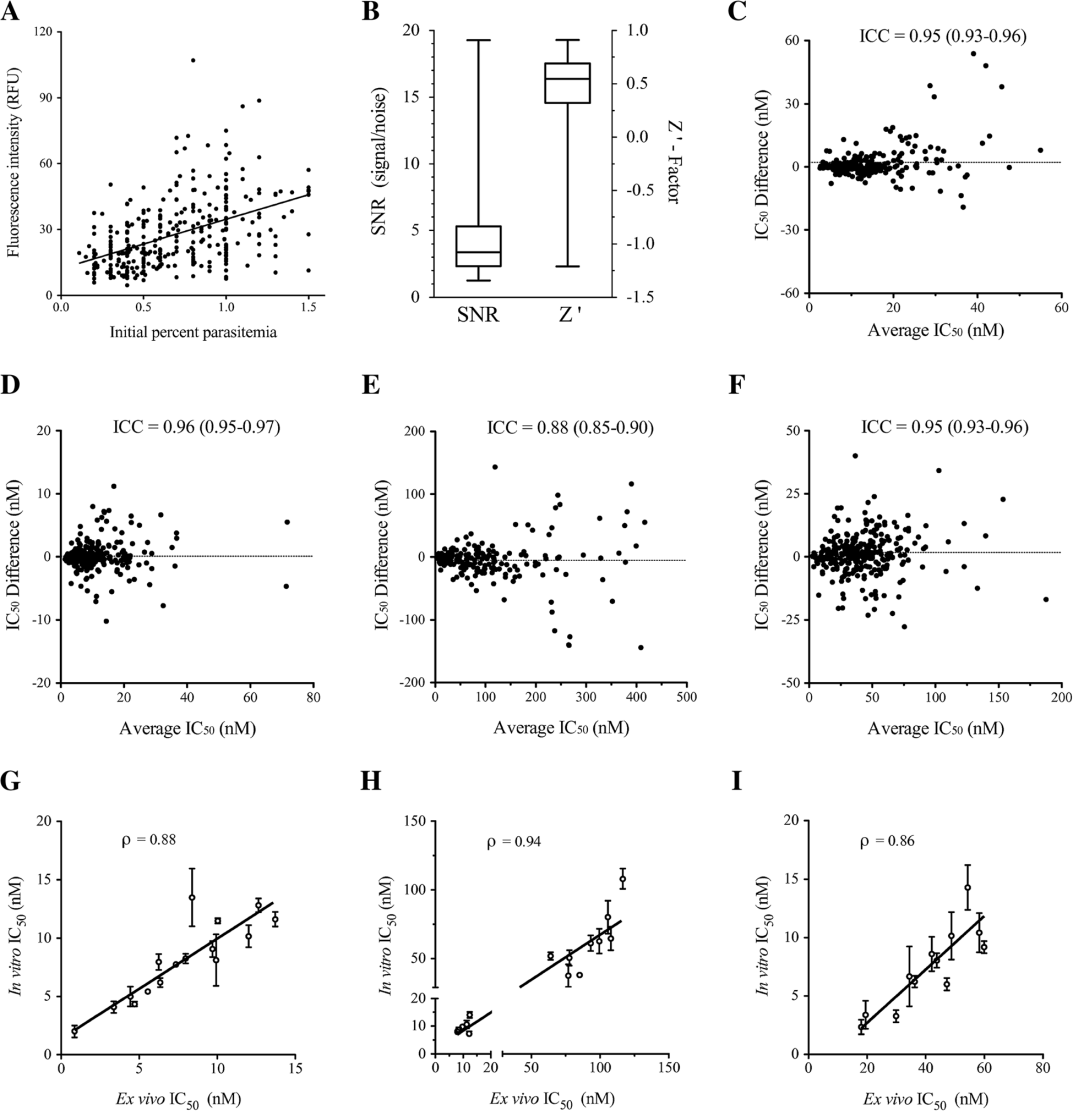
**Validation of the DAPI *ex vivo *drug assay. A**. Fluorescence intensity of maximum growth wells versus initial parasitaemia for parasites tested in the DAPI *ex vivo* drug assay. Pearson ρ = 0.47, linear slope *P* < 0.0001. **B**. Box plots showing signal-to-noise ratio (SNR) and Z’-factor for all assays. **C-F**. Bland-Altman plots showing differences between IC_50_ values of each technical replicate vs. average IC_50_ values for amodiaquine **(C)**, artemisinin **(D)**, chloroquine **(E)**, and mefloquine **(F)**. Horizontal lines indicate the mean difference in IC_50_ values between replicates. Intra-class correlation coefficients (ICC, with corresponding 95% confidence intervals) are displayed on each graph. **G-I.** Comparison of *ex vivo* with *in vitro* IC_50_ values for artemisinin **(G)**, chloroquine **(H)**, and mefloquine **(I)**, among culture-adapted monoclonal parasites collected in 2009. Mean *in vitro* IC_50_ values are plotted with error bars showing the standard error of at least two biological replicates. ρ denotes the Pearson correlation coefficient.

The usefulness of the DAPI-based *ex vivo* assay for monitoring drug sensitivity among the parasites circulating in Thiès, Senegal was assessed by measuring the dynamic range and reliability of the assay. This *ex vivo* assay could be used to accurately calculate anti-malarial 50-percent inhibitory concentrations (IC_50_ values) in direct patient samples, as evidenced by the dynamic range of the assay (Figure 1A and B), and by testing for reproducibility between technical replicates (Figure 1C-F). A subset of parasites derived from monoclonal infections was culture adapted and retested for their susceptibility to chloroquine, mefloquine and artemisinin *in vitro* (Figure 1G-I). *In vitro* drug responses were highly correlated with *ex vivo* responses, with systematic differences in scale presumably due to the technical differences between assays. Amodiaquine was not included in the *in vitro* testing because all parasites tested were sensitive to the drug, and the range of observed IC_50_ values was narrow. Overall, the DAPI *ex vivo* assay provided valid and consistent results, and could, therefore, be used as a tool to directly measure malaria parasite drug responses in patient samples.

### Distribution of parasite drug responses across years

**Figure 2:**
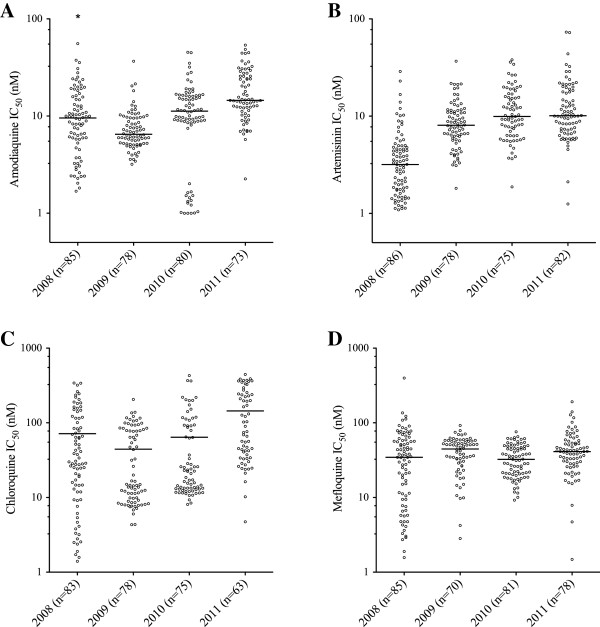
**Changes in *ex vivo *parasite sensitivity over time.** IC_50_ values among parasites collected in Thiès, Senegal and tested against amodiaquine **(A)**, artemisinin **(B)**, chloroquine **(C)**, and mefloquine **(D)**. The number of samples tested each year is indicted in parentheses below each plot. Horizontal lines indicate median IC_50_ values. The asterisk in panel **A** indicates an IC_50_ value off the scale (amodiaquine IC_50_ = 1140 nM).

The DAPI *ex vivo* drug assay was used to compare parasite drug responses to amodiaquine, artemisinin, chloroquine and mefloquine among all parasites tested between 2008 and 2011 (Figure 2, Table 2). During this time, parasite IC_50_ values increased for amodiaquine, artemisinin and chloroquine (*P* < 0.001 for linear and non-linear trends and in both crude and adjusted analyses). Parasite IC_50_ values for mefloquine also changed, but after adjusting for confounders only a non-linear trend in drug responses over time was detected. The 90^th^ percentile IC_50_ values, representing the most resistant parasites observed each year, also increased between 2008 and 2011 for amodiaquine, artemisinin and chloroquine (Table 2). Finally, there was a large range of parasite responses to amodiaquine and artemisinin, two anti-malarials that have been used in combination therapies in this area since 2006 [17]. IC_50_ values ranged from 1 nM to over 50 nM for amodiaquine (except for one outlier in 2008), and from 1 nM to over 70 nM for artemisinin.

**Table 2:** **Parasite drug responses measured in the DAPI *ex vivo *** **assay**

	**2008**	**2009**	**2010**	**2011**
	**(N = 86)**	**(N = 78)**	**(N = 81)**	**(N = 82)**
Amodiaquine				
Median IC_50_	9.6	6.5	11.2	14.5
IC_50_ Range	1.6, 1140	3.2, 36.7	1.0, 45.3	2.3, 53.7
90^th^ percentile IC_50_	24.0	11.8	22.2	35.4
Artemisinin				
Median IC_50_	3.2	8.1	9.9	10.1
IC_50_ Range	1.1, 28.8	1.8, 36.7	1.9, 38.0	1.3, 73.1
90^th^ percentile IC_50_	9.3	17.0	22.4	24.9
Chloroquine				
Median IC_50_	30.7	15.0	22.4	76.1
IC_50_ Range	1.4, 341.5	4.3, 205.5	8.1, 430.2	4.7, 455.0
90^th^ percentile IC_50_	199.4	108.2	199.1	364.6
Mefloquine				
Median IC_50_	34.5	44.6	32.6	41.2
IC_50_ Range	1.6, 398.0	2.8, 92.1	9.2, 75.7	1.5, 191.5
90^th^ percentile IC_50_	84.6	62.5	59.0	82.4

IC_50_ values are in nM. IC_50_ Range = minimum IC_50_, maximum IC_50_. 90^th^ percentile IC_50_ = IC_50_ value of the 90^th^ percentile within the range of drug responses observed each year.

### Prevalence of resistance-associated mutations over time

**Figure 3:**
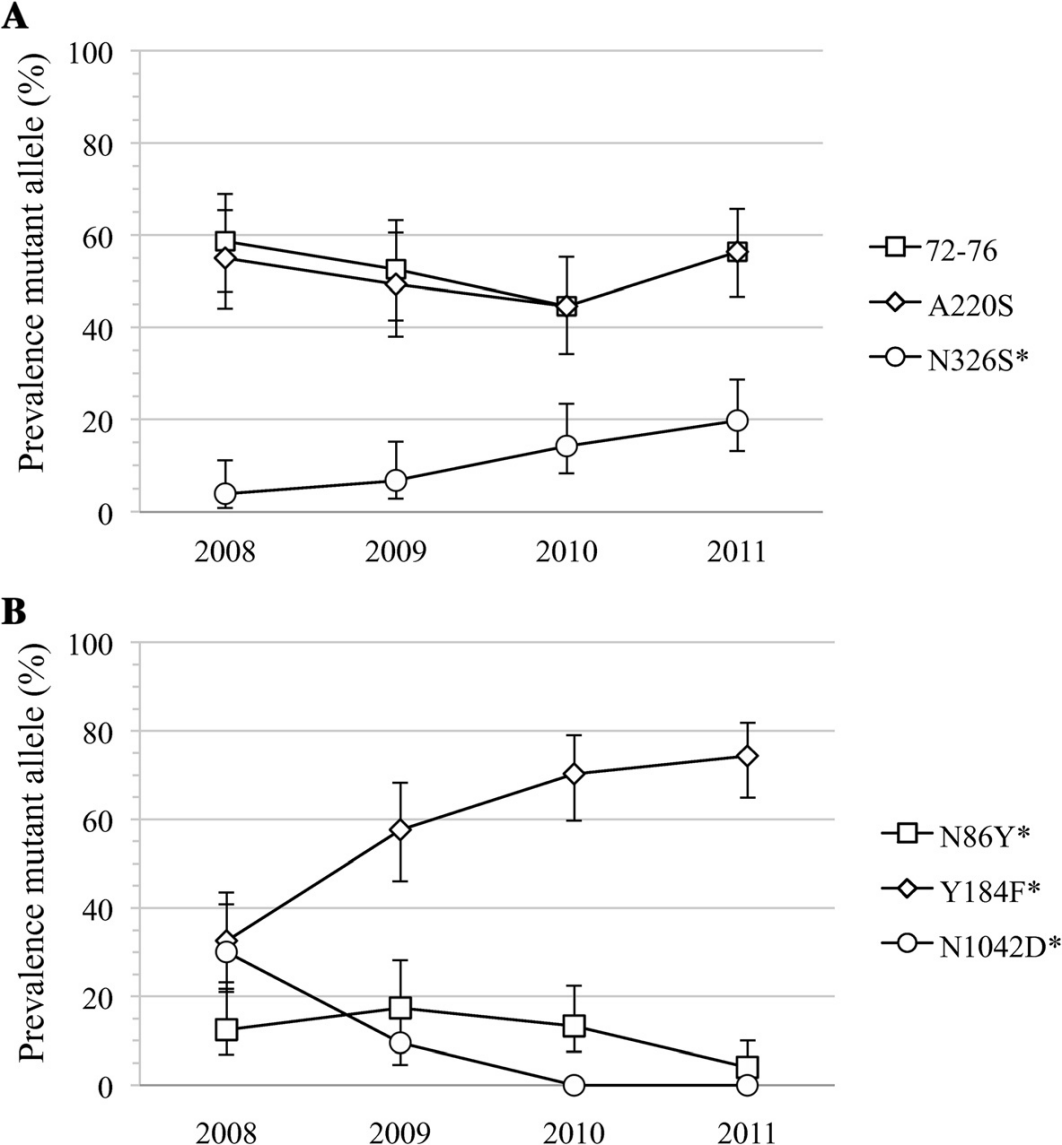
**Changes in prevalence of known drug resistance-associated mutations over time.** Prevalence of resistance-associated mutations in *pfcrt***(A)** and *pfmdr1***(B)** with corresponding 95% point-wise confidence intervals. Mutations were measured by high-resolution melt (HRM) technology and prevalence was calculated by dividing the number of samples containing at least one mutant allele by the total number of samples genotyped each year. Asterisks indicate significant changes over time (*P* < 0.05 by Fisher-Hamilton exact test).

In addition to directly testing parasite drug responses, known drug resistance-associated mutations in *pfcrt* and *pfmdr1* were genotyped in order to assess changes in the prevalence of these mutations over time. No change in the prevalence of the mutant haplotype at protein positions 72-76 within *pfcrt* was detected (Figure 3A). This haplotype was almost always inherited along with the A220S mutation in *pfcrt*, and both mutations remained above 50% prevalence in this population, in contrast to other studies within Senegal [20], and elsewhere in Africa [25]. Prevalence of the N326S mutation, however, did change over time (*P* < 0.05), and was not linked to the other typed mutations within *pfcrt*.

While the mutations typed in *pfcrt* either stayed the same or increased in prevalence over time, the mutations typed in *pfmdr1* showed two distinct patterns in their prevalence over the four years studied (Figure 3B). The N86Y and N1042D mutations both decreased in prevalence between 2008 and 2011 (*P* < 0.05). By 2011, the N86Y mutation was detected in fewer than 5% of parasites, and the N1042D mutation was absent from the population sampled in both 2010 and 2011. Conversely, the Y184F mutation more than doubled in prevalence, from approximately 30% in 2008 to greater than 70% in 2011 (*P* < 0.05).

### Correlations between resistance mutations and ex vivo drug responses

To assess whether the use of amodiaquine and artemisinin derivatives could be driving the observed changes in resistance mutation prevalence, associations between mutations and IC_50_ values were examined. The occurrence of mutant genotypes at *pfcrt* 72-76, A220S, and N326S were all associated with increased amodiaquine IC_50_ values (Table 3). These same *pfcrt* mutations were also associated with higher chloroquine IC_50_ values, possibly due to cross-resistance between chloroquine and amodiaquine (Pearson ρ = 0.6). The mutant 72-76 haplotype and A220S were also associated with artemisinin sensitivity, and none of the typed *pfcrt* mutations were associated with mefloquine response.

**Table 3:** **Associations between wild-type and mutant genotypes and *ex vivo *** **drug responses**

	**Median IC**_**50 **_**Values**											
	**(Interquartile Range)**											
	**Amodiaquine**			**Artemisinin**			**Chloroquine**			**Mefloquine**		
**Allele**	**Wild-type**	**Mutant**	***P***	**Wild-type**	**Mutant**	***P***	**Wild-type**	**Mutant**	***P***	**Wild-type**	**Mutant**	***P***
**N**_**(wt)**_**/N**_**(mut)**_												
*pfcrt* 72-76^a^	**9**	**12**	**0.004**	**9**	**7**	**0.005**	**14**	**99**	**< 0.0001**	42	36	0.3
160/182	**(6,15)**	**(8,19)**		**(6,15)**	**(4,10)**		**(11,28)**	**(53,196)**		(21,57)	(21,52)	
*pfcrt* A220S	**9**	**12**	**0.0005**	**9**	**7**	**0.01**	**14**	**99**	**< 0.0001**	42	36	0.3
163/174	**(6,14)**	**(8,19)**		**(6,15)**	**(4,10)**		**(11,28)**	**(53,196)**		(21,57)	(21,52)	
*pfcrt* N326S	**10**	**15**	**0.01**	8	9	0.6	**28**	**139**	**< 0.0001**	38	38	0.7
295/40	**(7,16)**	**(9,20)**		(5,13)	(5,12)		**(13,93)**	**(84,217)**		(21,54)	(28,56)	
*pfmdr1* N86Y	10	9	0.6	**8**	**5**	**0.0002**	33	75	0.4	**41**	**17**	**< 0.0001**
296/37	(7,16)	(6,17)		**(5,13)**	**(3,8)**		(14,101)	(12,201)		**(26,57)**	**(9,22)**	
*pfmdr1* Y184F	10	11	0.1	**7**	**8**	**0.01**	29	34	0.6	41	36	0.3
136/202	(6,16)	(7,16)		**(4,11)**	**(6,14)**		(12,109)	(13,114)		(21,58)	(21,52)	
*pfmdr1* N1042D	10	8	0.1	**8**	**4**	**<0.0001**	33	47	1.0	38	38	0.9
307/31	(7,16)	(5,16)		**(6,13)**	**(2,5)**		(13,109)	(15,115)		(21,53)	(18,59)	

IC_50_ values are in nM. N_(wt)_ = number of samples possessing only the wild-type allele. N_(mut)_ = number of samples possessing at least one mutant allele. Mixed genotypes were detected at the following prevalence: *pfcrt* 72-76 (n = 22); *pfcrt* A220S (n = 19); *pfcrt* N326S (n = 4); *pfmdr1* N86Y (n = 7); *pfmdr1* Y184F (n = 22); *pfmdr1* N1042D (n = 1). Genotypes were determined by high-resolution melt (HRM) genotyping. *P*-values were calculated using the Wilcoxon rank-sum test, and associations where *P* < 0.05 are shown in bold.

^a^*pfcrt* protein positions 72-76 were all perfectly correlated and were analysed as a haplotype rather than individually.

Associations between mutations in *pfmdr1* and parasite drug response were also detected. Parasites with wild-type alleles at amino acid positions 86 and 1042, as well as parasites with the mutant allele at position 184, had increased artemisinin IC_50_ values (Table 3). Artemisinin was the only drug associated with the Y184F mutation in this population – this mutation was not associated with amodiaquine, chloroquine, or mefloquine responses. No significant association was seen between amodiaquine or chloroquine responses and any of the mutations typed in *pfmdr1*, in contrast to previous findings [26-28], and perhaps due to a small number of parasites possessing the N86Y and N1042D mutations. Stratifying first on the *pfcrt* 72-76 haplotype did not reveal an association between amodiaquine or chloroquine and any of the *pfmdr1* mutations that were genotyped. Finally, higher mefloquine IC_50_ values were also associated with the wild-type allele at position 86 within *pfmdr1*; this was the only significant association between any of the typed mutations and mefloquine IC_50_ values.

## Discussion

*Ex vivo* assays are an important tool for malaria drug resistance monitoring in direct patient samples. These assays complement *in vivo* studies by allowing researchers to test parasite responses to different drugs individually and in the absence of patient factors that might introduce noise or confound results. Importantly, *ex vivo* monitoring of malaria parasite drug responses can provide an early warning of decreased parasite sensitivity before parasites become highly resistant and cause infected patients to fail drug treatment.

The DAPI *ex vivo* assay performed well, with excellent agreement between technical replicates, good dynamic range, and very good correlation between drug responses measured *ex vivo* with those measured *in vitro*. Furthermore, the IC_50_ values observed in Thiès, Senegal between 2008 and 2011 were comparable to other *ex vivo* studies of *P. falciparum* drug response [10,18,29]. Because parasite drug responses were measured over a four-year time span, trends in drug response in this population over time could also be assessed. The trends observed in parasite responses to amodiaquine and artemisinin suggest that malaria parasites in Thiès are becoming more tolerant to these compounds. Data from future years of *ex vivo* monitoring will be critical in determining whether these trends continue.

The trends observed in the prevalence of resistance-associated mutations in *pfcrt* and *pfmdr1* suggest that anti-malarial drug use is selecting for resistance-associated alleles within this population. In contrast to other studies [11,20,25], resistance-associated mutations within *pfcrt* remained prevalent within this population, and even appeared to increase in prevalence between 2010 and 2011. This suggests either that compensatory mutations have restored the fitness of resistant parasites, and/or that anti-malarial drug use is maintaining these mutations within the population. The continuous distribution of chloroquine IC_50_ values observed in 2008 and 2011 further suggests that additional mutations affecting parasite drug response exist in this population. The finding that parasites with mutations in *pfcrt* have higher amodiaquine IC_50_ values is in agreement with previous studies of laboratory parasite lines [26], and malaria-infected patients [28]. Additionally, amodiaquine has been administered to malaria-infected patients in Senegal since 2003 [17]. Because increased amodiaquine IC_50_ values were associated with the typed *pfcrt* mutations, it seems possible that use of amodiaquine is preserving these mutations within the population.

The observed trends in resistance mutations within *pfmdr1* suggest that artemisinin compounds are selecting for a combination of wild-type and mutant alleles within this gene. The N86 and 184F alleles have been previously associated with *in vivo* selection by ACT [30,31], and two recent studies of the prevalence of drug resistance markers in Dakar, Senegal also found a high prevalence of the Y184F mutation [32,33]. Furthermore, Y184F has been found to be under selection among parasite populations in Cambodia [34], where artemisinin resistance, defined as delayed parasite clearance, has been described. While the artemisinin resistance phenotype of delayed *in vivo* parasite clearance does not appear to correlate well with *ex vivo* or standard *in vitro* assays [2], artemisinin resistance might occur through different mechanisms in Africa as compared to southeast Asia. Furthermore, as parasites become increasingly artemisinin resistant *in vivo*, they may become amenable to monitoring with *ex vivo* assays such as this one. The disappearance of the N86Y and N1042D mutations, coupled with the rapid rise of the Y184F mutation, suggest that selective pressure is acting on *pfmdr1*, eliminating some mutations while driving others to high prevalence within this population. Because artemisinin response was associated with all three of the typed *pfmdr1* mutations, it appears that the artemisinin derivatives used in ACT might be the selective force driving the Y184F mutation to high prevalence, while simultaneously selecting for the wild-type alleles at positions 86 and 1042.

These findings are consistent with the hypothesis that amodiaquine use in Thiès, Senegal has selected for chloroquine resistance-associated mutations within *pfcrt*, while artemisinin compounds have selected for a particular combination of wild-type and mutant alleles within *pfmdr1*. In both cases, alleles that make parasites better able to withstand drug pressure are likely selected. Other African countries that have used artesunate-amodiaquine have also documented sustained high prevalence of chloroquine resistance-associated mutations within *pfcrt*[35,36]. Conversely, countries deploying ACT that does not include amodiaquine have seen a return to chloroquine-sensitivity after chloroquine was removed from the treatment arsenal [25,37], presumably due to the fitness costs of resistance-associated mutations. Other African countries have also documented recent increases in the prevalence of the *pfmdr1* N86 and 184F alleles [38,39], though this study marks the highest recorded prevalence to date of the Y184F mutation on the African continent.

## Conclusions

*Ex vivo* monitoring of malaria parasite drug response is a powerful tool for malaria control. Directly testing parasite drug responses and genotyping resistance-associated mutations can provide early warnings of decreased parasite sensitivity to the individual drugs used in combination therapies, before parasites become highly resistant and cause infected patients to fail drug treatment. *Ex vivo* drug assays such as the one used here could also provide phenotype data for analyses aimed at identifying additional drug resistance markers [40-42]. As ACT is the first-line treatment throughout Africa, the ability to detect resistance to both artemisinin derivatives and partner compounds as they emerge will become increasingly important in order to preserve the efficacy of these drugs.

## Competing interests

The authors declare that they have no competing interests.

## Authors’ contributions

DVT, PDS, ON, SM, SKV, DFW and DN designed the experiments. DVT, BD, RFD, PDS, AKL, MN, AKB, YDN, EJH and SKV carried out the experiments and collected data. DVT, RFD, CV, PDS and SKV analysed the data. DVT, CV, SKV, DFW and DN wrote the manuscript. All authors read and approved the final manuscript.

## Authors’ information

Daria Van Tyne and Baba Dieye are equal contributors (co-first authors).

Clarissa Valim and Rachel F Daniels are equal contributors (co-second authors).
